# Porcine Aorto-Renal Artery (PARA) model for laparoscopic transcystic common bile duct exploration: the evolution of a training model to meet new clinical needs

**DOI:** 10.1007/s00423-020-02045-0

**Published:** 2021-02-17

**Authors:** James O. Brewer, Lalin Navaratne, Stephen W. Marchington, David Martínez Cecilia, Jose Quiñones Sampedro, Luis Muñoz Bellvis, Alberto Martínez Isla

**Affiliations:** 1 Department of Upper Gastrointestinal Surgery, Northwick Park Hospital, London North West University Healthcare NHS Trust, Watford Road, Harrow, London, HA1 3UJ UK; 2 Defence Medical Services, Birmingham, UK; 3 Department of Surgery and Cancer, Imperial College London, London, UK; 4 Hospital Universitario de Toledo, Toledo, Spain; 5 Hospital Universitario de Salamanca, Salamanca, Spain

**Keywords:** Choledocholithiasis, Common bile duct stones, Laparoscopic common bile duct exploration, Laparoscopic transcystic common bile duct exploration, Porcine Aorta-Renal Artery model

## Abstract

**Background:**

The transcystic approach to laparoscopic common bile duct exploration has gained popularity for the single-stage management of choledocholithiasis with concomitant gallstones. Our team previously described the use of a porcine aorta segment to simulate the common bile duct during laparoscopic skill training.

**Methods:**

With the advent of the transcystic approach as a contender for the first-line technique of accessing the common bile duct, we present an evolution of the laparoscopic training model using a Porcine Aorta-Renal Artery (PARA) specimen to simulate the structural integrity, dimensions and spatial distribution of both the human cystic and common bile ducts.

**Results:**

This training model allows the use of a choledochoscope for transcystic exploration of the biliary tree. It combines fidelity and reproducibility required for a simulated training model to offer experience in laparoscopic transcystic common bile duct exploration. Validation of the model was demonstrated by 21 surgeons who completed a questionnaire after performing the simulated procedure. In all sections assessing reliability, face validity and content validity of the model, mean rating scores were between 4 and 5 out of five (good or excellent).

**Conclusions:**

We present the evolution of an established training model for laparoscopic common bile duct exploration which focusses the attention on the transcystic approach to the common bile duct and the use of lithotripsy techniques. The need for such a model reflects the shift in the current practice of the laparoendoscopic management of choledocholithiasis with concomitant gallstones from transductal to transcystic approach.

**Supplementary Information:**

The online version contains supplementary material available at 10.1007/s00423-020-02045-0.

## Introduction

Clearance of common bile duct (CBD) stones by laparoscopic common bile duct exploration (LCBDE) at the time of cholecystectomy is the preferred treatment for choledocholithiasis with concomitant gallstones, provided that the necessary expertise are available [1]. More recently, with the development of smaller choledochoscopes and stone fragmentation techniques, the transcystic approach to the common bile duct facilitating choledochoscopic stone extraction has gained popularity [2, 3].

Ricci et al. reported on the efficacy and safety of laparoscopic and endoscopic techniques for the management of common bile duct stones using a network meta-analysis [4]. Their systematic review included 20 randomised studies, with 14 of these comparing LCBDE with other endoscopic techniques such as preoperative, intraoperative or postoperative endoscopic retrograde cholangiopancreatography (ERCP). The pooled number of patients undergoing LCBDE included in their study was 915; however, the authors found that only 331 (36.2%) cases used the transcystic approach to explore the CBD. Reinders et al. published a systematic review on transcystic versus transductal stone extraction during single-stage treatment of choledocho-cysto-lithiasis in 2014 [5]. The authors reported more bile leaks and overall morbidity after transductal approach when compared with transcystic stone extraction (11% vs 1.7%, *p* < 0.05 and 18.4–26.7% vs 7–10.5%, *p* < 0.05 respectively). Pang et al. more recently published their results from a similar study and found that transcystic exploration had significantly shorter operative time and hospital stay, less operative blood loss and fewer complications, and was more cost-efficient than traditional laparoscopic common bile duct exploration [6]. Adjuncts such as holmium laser lithotripsy and electrohydraulic lithotripsy further increase the success rate of transcystic LCBDE [7]. At the authors institution (JB, LN and AI), holmium laser lithotripsy has increased the rate of transcystic LCBDE from 67 to 83% within the last 5 years (179 patients) [8]. Technological advances in flexible 3-mm choledochoscopy will likely contribute to incremental gains in the success of transcystic CBD exploration. The aim of this paper is to describe a reproducible and realistic training model for laparoscopic transcystic common bile duct exploration.

## The evolution of a training model

Increasing popularity of minimally invasive techniques has motivated the surgical community to develop in-house training models to simulate and teach the laparoscopic approach to CBD exploration. However, LCBDE may be an underused treatment for choledocholithiasis due to the lack of exposure to the procedure during surgical training (residency). A study examining operative logbooks demonstrated that residents performed a mean of only 0.7 LCBDEs during their entire training [9]. Sánchez et al. and Santos et al. have presented training models for LCBDE, which are constructed from available medical devices such as urinary catheters and latex tubes [10, 11]. These models have been used in training protocols, receiving good trainee feedback and achieving desirable training goals (content validity). The authors demonstrated that using a LCBDE curriculum improved the ability of surgeons to perform both transcystic and transductal LCBDE on a procedural simulator [12]. Furthermore, this simulation-based mastery-learning curriculum increased institutional utilisation of LCBDE at the authors institution [13]. These models are cost-effective and provide reproducible training scenarios (reliability); however, they lack realistic tissue-handling experience (face validity). The first virtual reality protocol with haptic feedback for common bile duct exploration was described in 2001 by Basdogan et al.; however, to date there is no commercially available virtual reality simulator for training in common bile duct exploration [14]. The use of an animal model allows the trainee to visualise the effects of manipulation and surgical instrumentation on the tissues. Furthermore, animal models also enable training in the use of energy devices, surgical devices (e.g. clip applicator) and various forms of lithotripsy (e.g. holmium laser lithotripsy and electrohydraulic lithotripsy). However, animal models are not reusable and require expertise and infrastructure for their preparation and adequate storage, with associated additional running costs for the training facilities. From the ethical perspective, within the UK, the use of animal models is regulated by the Animal By-Products (Enforcement) (England) Regulations 2011. It is the training facility’s responsibility to obtain organic specimens from approved sources.

Our team previously described the use of segmental porcine aorta to simulate the CBD [15]. This model was affordable, reproducible and well-received for training in transductal LCBDE. The diameter and consistency of the porcine aorta resembles that of the human CBD, making it ideal for training in laparoscopic surgical skills and flexible choledochoscopy. This model has been successfully implemented by the Pan-London Surgical Skills Training Programme as part of the curriculum provided to all higher specialist trainees with an interest in upper gastrointestinal surgery. With the advent of the transcystic approach as a contender for the first-line technique in CBD exploration, we present an evolution of the laparoscopic training model using an animal specimen that would include the porcine aorta and the right renal artery to mimic the cystic duct. The porcine aorto-renal artery (PARA) model for CBD exploration offers very similar dimensions and spatial distribution to both the human cystic and common bile ducts.

## Description and preparation of the model

**Fig. 1:**
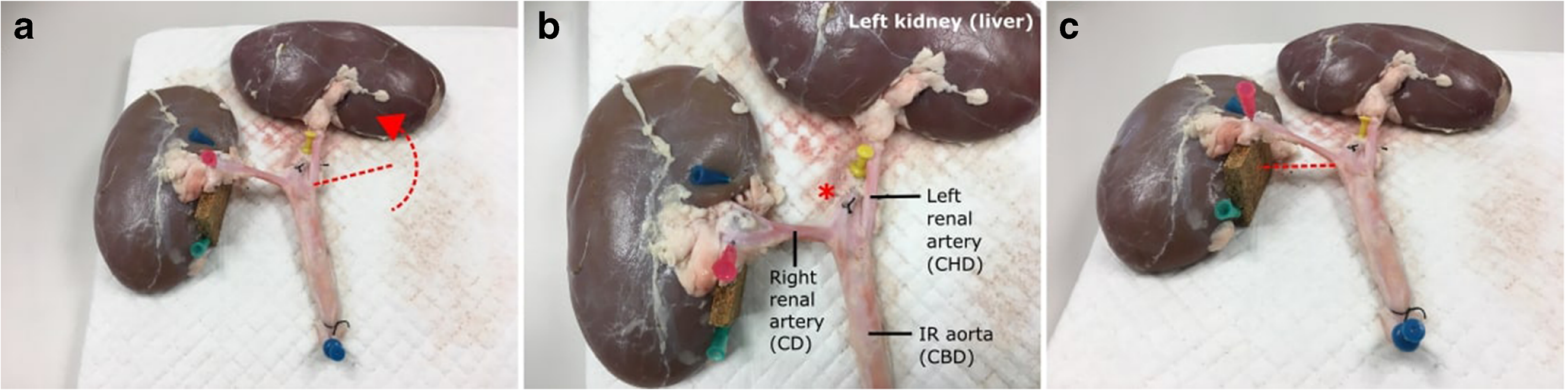
Preparation of the model. **a** Anti-clockwise rotation of the left renal artery and kidney. **b** The suprarenal aorta is ligated at the level of the renal arteries (brackets = simulated anatomical structure). **c** The right renal artery is raised by placing a 40-mm piece of cork board under the hilum of the right kidney to simulate the in vivo angle of the cystic duct related to the common bile duct. CD, cystic duct; CHD, common hepatic duct; IR, infrarenal; CBD, common bile duct; *ligation of the suprarenal aorta

The porcine renal system is used en bloc, comprising the aorta, vena cava, renal vessels and kidneys. The organs, obtained as a by-product of farming, are collected from pigs weighing approximately 60 kg. The aorta, renal artery and kidney are placed on a cork board (Fig. 1). Minimal dissection is required to isolate the required vessels. The left kidney and left renal artery are rotated 90° anti-clockwise about its vascular pedicle to lie in a position which simulates the liver and common hepatic duct respectively (Fig. 1a). The suprarenal aorta is then ligated flush with the level of the renal arteries (Fig. 1b*). The distal aorta is partially ligated to simulate the duodenal papilla. To better simulate the anatomical configuration between the cystic duct and the CBD, the distal right renal artery is raised approximately 40 mm from the flat surface of the cork board, allowing it to join the aorta at an angle similar to the human cystic duct (Fig. 1c).

**Fig. 2:**
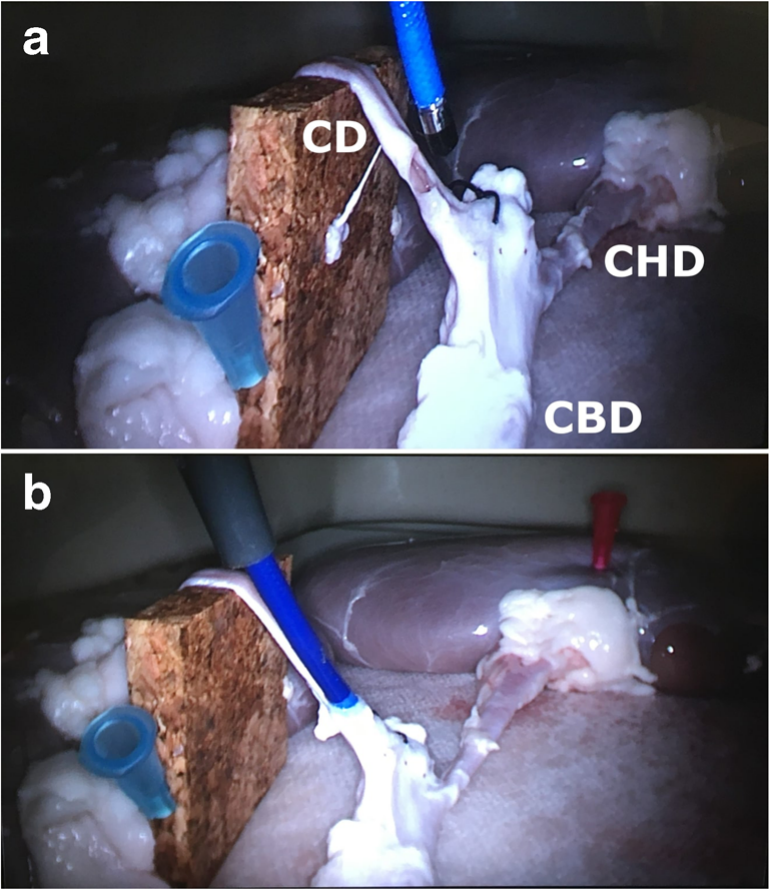
Laparoscopic transcystic common bile duct exploration using a 3-mm choledochoscope. **a** Cannulation of the cystic duct. **b** Choledochoscopy (distal view of common bile duct). CD, cystic duct; CHD, common hepatic duct; CBD, common bile duct

**Fig. 3:**
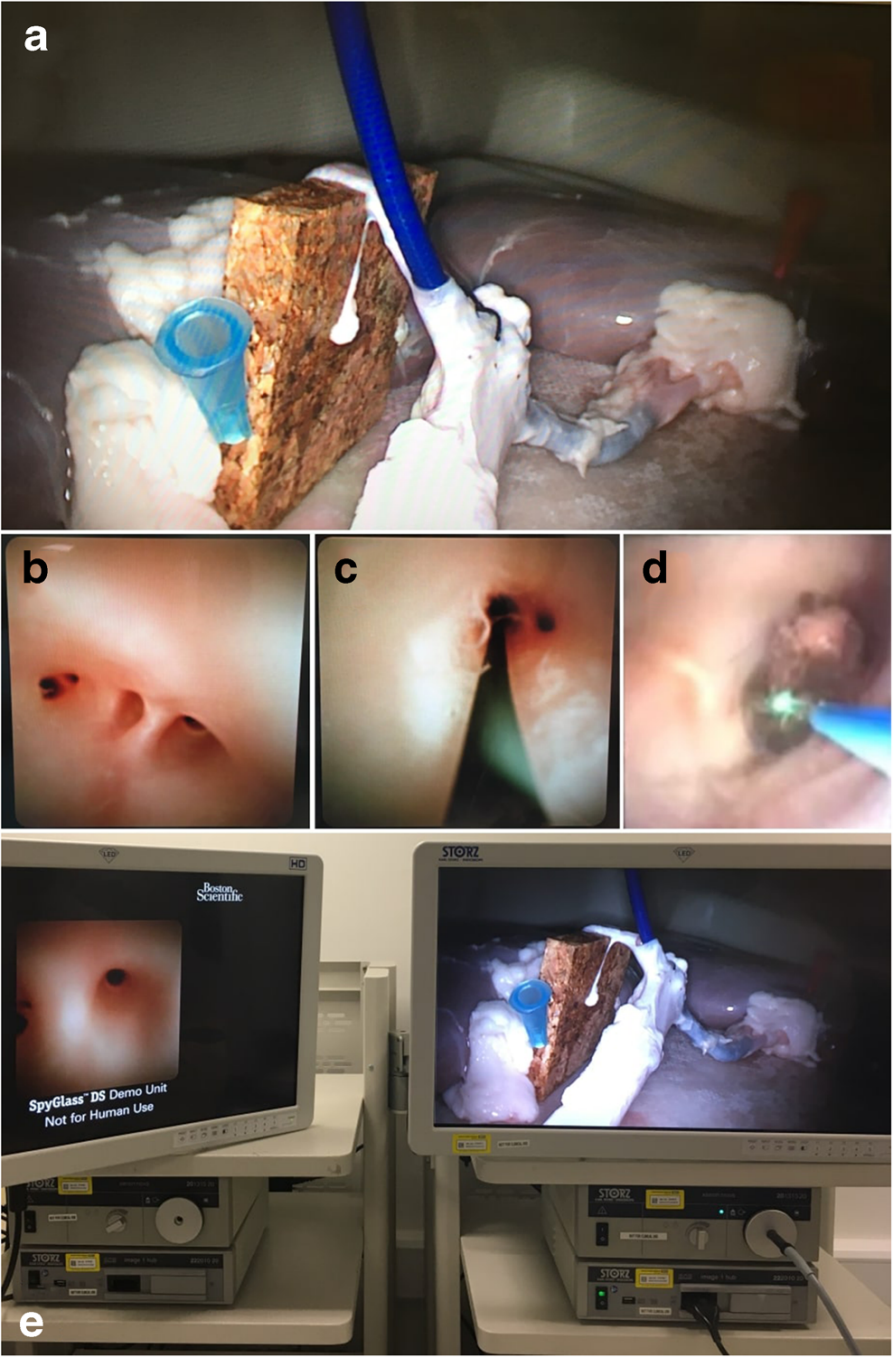
Proximal choledochoscopy using a 3-mm choledochoscope. **a** Laparoscopic view of proximal CBD cannulation. **b** Accompanying choledochoscopic view of left kidney hilum simulating intrahepatic ducts. **c** Selective cannulation of an intrahepatic duct with guidewire. **d** Stone fragmentation with holmium laser lithotripsy (HLL). **e** Simulation setup with monitor for choledochoscopy (left) and laparoscopic monitor (right)

Once the model is prepared, a few chickpeas or pepper seeds are introduced into the lumen of the aorta to simulate CBD stones. The distal end of the aorta is partially open to allow drainage of saline if a wet model with irrigation is used. This is necessary if electrohydraulic lithotripsy or holmium laser lithotripsy is used within the model to train stone fragmentation techniques. The prepared model is then placed inside the laparoscopic training box and an extra 5-mm port or scope introducer is inserted into the right upper quadrant in the direction of the simulated cystic duct (Fig. 2). The simulated cystic duct is incised with laparoscopic scissors. At this stage, the trainee can choose to insert a cholangiography catheter, and then a guidewire to practice cystic dilatation manoeuvres followed by the insertion of a 3- or 5-mm choledochoscope. An insertion of a 5-mm choledochoscope requires dilatation of the simulated cystic duct as frequently seen in the operating theatre. The model reliably allows the delegates to perform ductal stone retrieval using Dormia baskets and perform proximal and distal cholangioscopy using the windscreen-wiper manoeuvre as described by Gough et al. (Fig. 3) [16]. Furthermore, trainees can practice advanced techniques such as lithotripsy techniques, laparoscopic suture ligation of a short cystic duct stump and the use of an Endoloop to ligate the simulated cystic duct at the end of the procedure.

Each prosection is acquired for £18.50 ($23.50) and is received and stored frozen. The model endures multiple attempts of cystic duct cannulation, CBD exploration and stone retrieval without losing structural integrity. The PARA model is currently being used by the Surgical Innovation Centre within the Department of Surgery and Cancer at Imperial College London, UK, as part of the London General Surgical Skills Programme for senior upper gastrointestinal trainees. The current model has also been adopted as the LCBDE training platform for courses in Spain (Salamanca and Toledo).

## Validation of the PARA model for laparoscopic transcystic common bile duct exploration

We validated this model using a questionnaire by assessing 5 criteria amongst 21 surgeons (Table 1). There were 16 consultants and 5 registrars (residents). All registrars were in a designated surgical training programme. There was a wide range of prior experience in LCBDE amongst the participating surgeons. Consultant surgeons (*n* = 16) had been appointed for a median of 7.5 years (range 1–15 years) and performed a mean of 40 LCBDE procedures (range 0–375). Surgical residents (*n* = 5) had been in their training programme for a median of 4 years (range 1–5 years) and assisted in a mean of 4 LCBDE procedures (range 0–7). Table 2 shows the validation scores amongst consultant and registrar surgeons. Each criterion was scored on a Likert scale from 1 (poor performance of the model) to 5 (excellent performance of the model). All surgeons consistently scored reliability, face validity and content validity highly with mean scores of 4.7, 4.5 and 4.8 respectively. There were no significant differences in scores according to seniority or experience. When participants were asked to rate their confidence of performing transcystic LCBDE in a live patient after using the model, scores were consistently above 4 out of five. As expected, consultants appeared to be more confident than registrars, confirming surgical experience and seniority as confounding factors.

**Table 1:** Validation questionnaire

Validation criteria	Question
Reliability	How reproducible do you think this model is?
Face validity	How realistic do you think this model is?
Content validity	How appropriate do you think this model is as a teaching modality?
Operator confidence	After using this model, how confident are you in performing transcystic LCBDE in a live patient?

*LCBDE*, laparoscopic common bile duct exploration

**Table 2:** Validation data amongst 21 participants using the PARA model for LCBDE training. Mean scores are presented. The validation criteria were scored from 1 (poor performance) to 5 (excellent performance)

	No. of participants	Reliability (1–5)	Face validity (1–5)	Content validity (1–5)	Operator confidence (1–5)
Consultant	16	4.6	4.6	4.7	4.3
Registrar	5	4.8	4.4	5	4.0
All participants	21	4.7	4.5	4.8	4.2

The main aim of this validation data was to provide evidence of reliability and face validity. There are limitations to the data presented here. The current validation data presented here reports reliability, face validity and content validity, rather than the Messick validity framework, considered the gold standard for evaluating training models [17]. We have not provided procedural times for completing stone extraction during the training sessions as the large number of surgeons with few simulators did not allow for multiple attempts for each participant. It was also not possible to compare performance in the operating theatre before and after use of this training model. Therefore, we are unable to provide evidence here that use of this model translates into enhanced performance in clinical practice. However, exposure to LCBDE during surgical training is very low, and the model described here is a reproducible and realistic training model which can be packaged into a LCBDE curriculum for higher surgical trainees prior to starting these procedures in live patients. There is evidence that the use of such a curriculum improved the ability of surgeons to perform both transcystic and transductal LCBDE on a procedural simulator and increased institutional utilisation of LCBDE [12, 13].

Future research should focus on validating this model using the Messick validity framework [17] and evaluating the benefits of using this model within a modular training curriculum. This could be assessed by a randomised study within the training setting, similar to the LapTrain multi-modality training curriculum for laparoscopic cholecystectomy [18]. A scoring system to assess such training modules can be developed, such as a ‘LCBDE-specific’ Objective Structured Assessment of Technical Skills (OSATS) global rating scale, to evaluate the skills of surgical trainees in the operating room [19].

## Conclusion

The PARA model is a reproducible and realistic training model for transcystic LCBDE. To our knowledge, this is the first description of an animal model for transcystic LCBDE. The model appears to be an excellent training platform, enabling users to gain experience in transcystic LCBDE. The model still allows for training in transductal LCBDE with choledochotomy as well as more recently developed lithotripsy techniques to fragment larger and/or impacted stones.

## Supplementary Information

ESM 1 (MP4 129980 kb).
